# Diagnostic role of urine human epididymis protein 4 in ovarian cancer

**DOI:** 10.11613/BM.2024.030502

**Published:** 2024-10-15

**Authors:** Antonija Hanžek, Christian Siatka, Anne-Cécile E. Duc

**Affiliations:** UPR CHROME - Nîmes University, Nîmes, France

**Keywords:** epididymis-specific protein E4, human, ovarian cancer, tumor markers, urine, diagnosis

## Abstract

Ovarian cancer is the 8th most common malignancy in women and the deadliest gynecological cancer. Due to the non-specific symptoms and the lack of effective diagnostic methods, late diagnosis remains the main barrier for improving the poor prognosis. Human epididymis protein 4 (HE4) is a protein overexpressed in ovarian cancer, but not in healthy individuals or benign conditions. The aim of this review article is to evaluate the laboratory aspect and potential clinical application of urine HE4. The methodology is presented, together with discussion on preanalytical, analytical and postanalytical phase of HE4 detection using urine. Moreover, we present the diagnostic role of urine HE4 in differential diagnosis, chemotherapy response, detection of recurrence and detection of low-malignant potential tumors. It has been found that urine HE4 presents as a promising, non-invasive tumor marker for detection and monitoring of ovarian cancer. However, standardization of the HE4 detection process is needed prior to implementation in clinical diagnostics.

## Graphical abstract

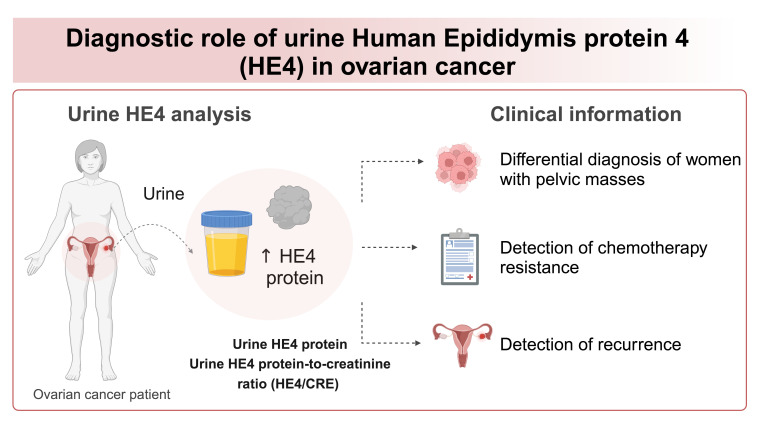

## Highlights

• Urine human epididymis protein 4 (HE4) and protein-to-creatinine ratio (HE4/CRE) are higher in ovarian cancer

• Urine HE4 and HE4/CRE ratio can be used in the differential diagnosis of ovarian cancer

• Urine HE4/CRE is a predictive marker of chemotherapy resistance and disease recurrence

• Urine HE4 can be detected in urine by immunoassays or mass spectrometry

• Standardization of the urine HE4 determination is needed before its clinical application

## Introduction

Ovarian cancer (OC) is the leading cause of death among all gynecological cancers (*1*). It is the 8^th^ most common cancer type and the 9^th^ cause of all cancer-related death (*1*-*4*). In the next decade, OC could become a public health problem, with a predicted 55% increase in global incidence and a 67% increase in mortality by the year 2035 (*5*). Ovarian cancer is a heterogeneous disease, with the majority (90%) of tumors arising from the surface epithelium, called the epithelial ovarian cancer (EOC) (*6*). The World Health Organization recognizes several distinct histological subtypes: serous, mucinous, endometrioid, clear cell, and transitional EOC (*7*, *8*). The rarer (10%) types are sex-cord stromal, germ cell or mixed cell types depending on the cell origin (*9*).

When diagnosed in early stages (stage I/II), 5-year survival rate is 90% (*10*). Unfortunately, two third of all cases are diagnosed in advanced stages (stage III/IV), when disease spread outside the pelvis, with a 5-year survival dropping to 15-40% (*8*, *10*). Due to non-specific symptoms (bloating, pelvic pain, weight loss, frequent need to urinate, fatigue) and lack of efficient diagnostic methods, late diagnosis remains a crucial hurdle to reduce the mortality (*6*). Standard diagnostic approach includes combination of the pelvic examination, transvaginal ultrasound and elevated serum concentration of cancer antigen 125 (CA125), but they are not sensitive nor specific enough to detect the disease in the early stage (*6*, *11*). Currently, no screening methods are available for OC (*12*). Moreover, the gynecological examinations can be considered invasive by some women, causing them to delay setting-up doctors’ appointments (*13*). Therefore, less invasive approaches could be beneficial to increase the participation in diagnostic procedures and improve the poor prognosis (*14*).

Urine has been studied as an alternative sample in detection of tumor markers (*14*-*16*). Human epididymis protein 4 (HE4) is a glycoprotein overexpressed in OC, but not in healthy individuals or benign conditions, and has clinically validated diagnostic value in serum (*17*-*20*). Recently, it has been shown that HE4 protein is also present in urine of OC patients (*20*). Therefore, the potential introduction of urine HE4 analysis in clinical diagnostics was proposed (*21*, *22*). Herein, we present the overview of the recent knowledge of the diagnostic role of urine HE4 with a focus on laboratory aspect of HE4 detection and its clinical application in management of OC. The aim of this article is to present the methodology, together with discussion on preanalytical, analytical and postanalytical phase of urine HE4 detection. Moreover, we present the diagnostic role of urinary HE4 in differential diagnosis, chemotherapy response, detection of recurrence and detection of low-malignant potential tumors.

## Literature research strategy

**Figure 1:**
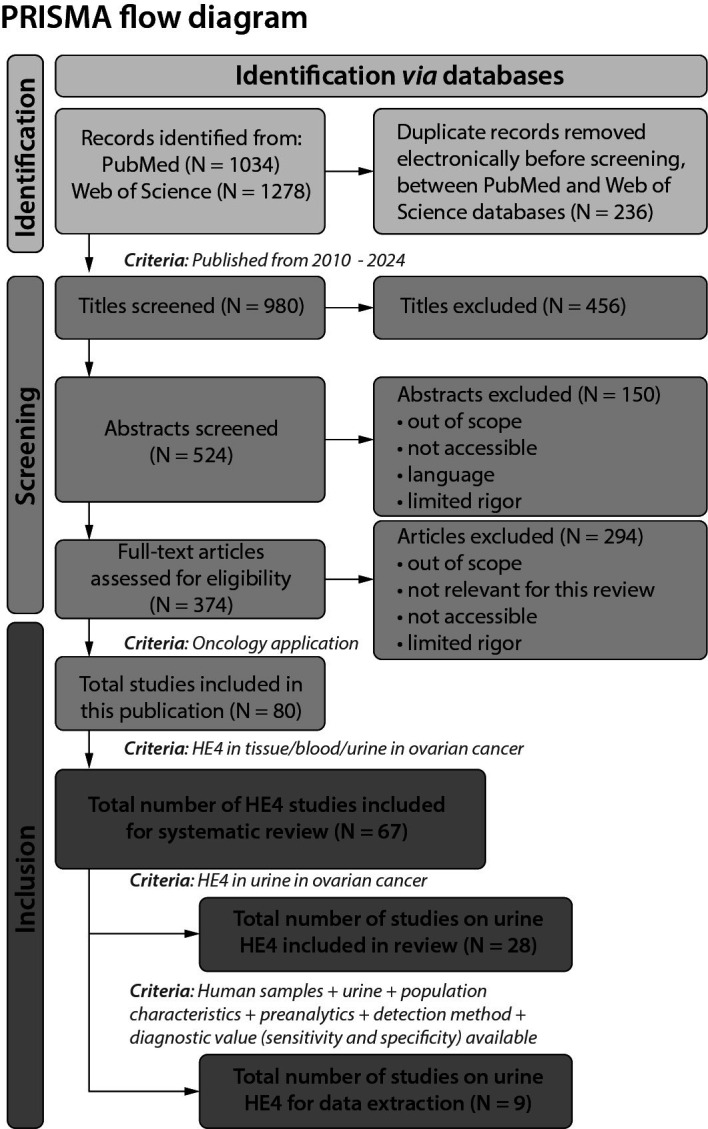
Flow diagram of the literature search. The search yielded 980 unique publications from PubMed and Web of Science databases. The retrieved records were first screened based on title and abstract, followed by full-text evaluation. Eighty studies were eligible for inclusion if they reported detection of human epididymis protein 4 (HE4) for oncology applications in tissue, blood or urine. From those articles, the 28 articles that focused specifically on HE4 in urine in ovarian cancer were eligible and are included in this review, and from 9 articles detailed data on urine HE4 methodology and clinical samples was extracted. Created with BioRender.com.

This review was performed with guidance from the general principles of the Preferred Reporting Items for Systematic Reviews and Meta-Analyses (PRISMA) statement (*23*). The search of the literature was performed using databases PubMed and Web of Science, using the keywords “Human Epididymis protein 4,” “HE4,” or “WFDC2 (*whey-acidic-protein four-disulfide core domain protein 2*)” combined with the terms “Ovarian Cancer/Cancer”, ”Blood/Serum/Urine”, “Tumor Marker”, “Diagnostic” or “Prognostic” published from a period from 2010 to 2024. The research yielded 980 unique hits. The retrieved records were first screened based on title and abstract, followed by full-text evaluation, resulting in the 374 articles. Studies were eligible for research if they reported on the use marker HE4 protein for oncology application and its related laboratory methodology, resulting in a total of 80 articles included in this publication. From those publications, 67 studies that reported on HE4 in tissue, serum or urine were selected for this study. The publications that specifically reported HE4 protein in urine resulted in total of 28 articles. From the 9 studies limited to human subjects (patients) that investigated urine HE4 in OC, the information related to: study population characteristics, urine sample, preanalytics, detection method, results interpretation, study conclusions, and the diagnostic value (sensitivity and specificity) was obtained. The flowchart of the research strategy is presented in Figure 1.

## Human epididymis protein 4 as a tumor marker of ovarian cancer

**Figure 2:**
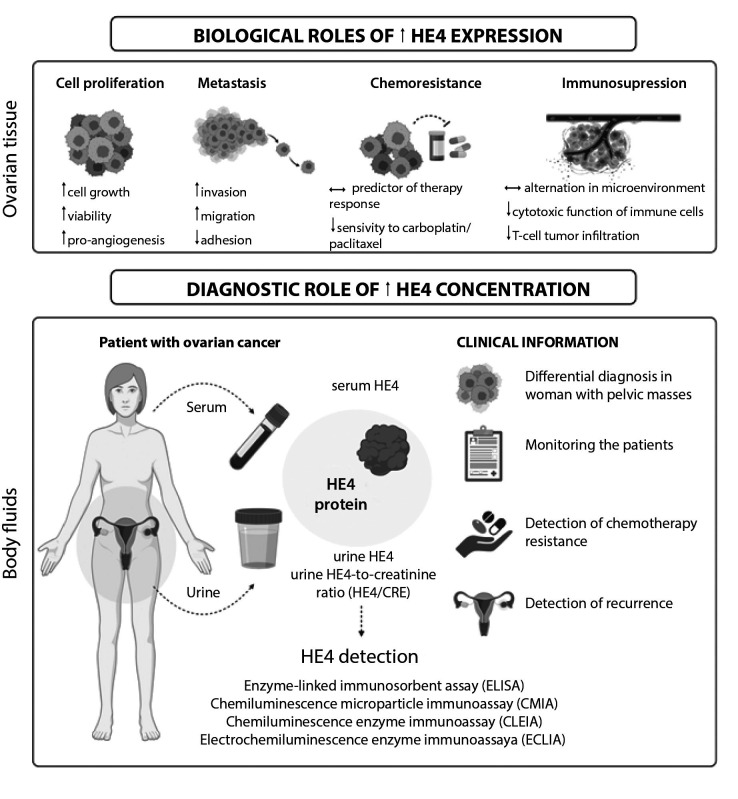
Human epididymis protein 4 in ovarian cancer. The overexpression of HE4 protein is involved in the pathogenesis of ovarian cancer and detected in serum and urine of patients. High concentrations of HE4 are used in diagnosis, monitoring the patients and for detection of recurrence. Created with BioRender.com.

Human epididymis protein 4 is a tumor marker used in differential diagnosis, prognosis and monitoring the patients with OC (*21*, *24*-*26*). It was first identified in 1991 in the distant epididymis, originally predicted as protease inhibitor involved in spermatogenesis (*27*, *28*). The HE4 protein is encoded in *HE4* or *WFDC2* gene, located on chromosome 20q12 (*29*). The gene encodes a small 11–25 kDa protein, also known as whey acidic protein (WAP) four-disulfide core domain protein 2, which belongs to the WAP domain family (*29*). Human epididymis protein 4 can have low expression in a normal tissues, including reproductive system, respiratory tract, nasopharynx and salivary glands (*30*). Since overexpression in OC tissue was discovered in 1999, HE4 is well known as a tumor marker for OC (*31*). It has been connected to oncogenic pathways that play roles in OC proliferation, metastatic potential, resistance to chemotherapy and immunosuppression (*32*-*35*). The biological roles of HE4 in the pathogenesis of OC are illustrated in Figure 2.

As a secreted protein, it is detected in body fluids of women with OC (*19*, *36*). Human epididymis protein 4 is elevated in serum (≥ 70 pmol/L) in all subtypes of OC (*37*). The highest concentration is present in serous, followed by mixed, endometrioid, clear cell and lowest in mucinous OC (*37*).

Diagnostic test aims to classify or predict the presence or absence of OC (*38*). The two metrics used to assess the diagnostic accuracy of tumor markers are sensitivity and specificity (*38*). Sensitivity is a test’s ability to identify OC when it is truly present and is given as a ratio of true positives/(true positives + false negatives) (*38*). Specificity is test’s ability to exclude OC in individuals who do not have the disease and is given by a ratio of true negatives/(true negative + false positives) (*38*). Numerous studies have reported that high concentration of serum HE4 have superior sensitivity and specificity than serum CA125 for detection of OC (*20*). Higher serum concentrations of CA125 (≥ 35 U/mL) is found in 50% of patients with stage I/II and 85% of patients with stage III/IV disease, limiting its utility in early detection (*39*, *40*). However, it has been reported that HE4 protein is increased earlier in the course of the disease (*39*, *40*). Moreover, HE4 offers advantages in terms of specificity, as CA125 is physiologically elevated in pregnancy, during menstruation and in benign gynecological conditions (ovarian cysts, endometriosis, pelvic inflammatory disease, *etc.*) (*41*). On the contrary, serum HE4 concentrations are decreased during and after pregnancy, can be measured at any phase of menstrual cycle or under contraception treatment (*42*-*44*).

The meta-analysis of more than 14,000 patients revealed that serum HE4 concentrations have combined sensitivity of 79% and specificity of 92% for differentiating OC from benign conditions and healthy individuals (*20*).

Higher concentrations of HE4 are present in individuals who are resistant to chemotherapy (*45*). Therefore, it has a predictive role in monitoring the therapy response (*36*, *46*). Moreover, HE4 concentration correlates with tumor burden, it is used for the detection of recurrence (*31*, *46*). Indeed, in 2008, the Food and Drug Agency (FDA) approved HE4 as an aid in monitoring recurrence or progressive disease in patients with epithelial OC (*31*). However, according to the guidelines by European Society for Medical Oncology and European Society of Gyneacological Oncology, serum HE4 testing alone is currently not recommended in routine assessment of therapy response and disease progression (*47*). To increase the diagnostic performance, combined use of different tumor markers should be considered (*48*). In 2011, the FDA approved the use of serum HE4 in combination with CA125 as part of Risk of Ovarian Cancer Malignancy Algorithm (ROMA), to determine the likelihood of malignancy in women presenting with adnexal mass (*49*). Sensitivity and specificity of ROMA is 74% and 89% in premenopausal woman, and 95% and 83% in postmenopausal, respectively (*50*). According to the guidelines by the European Group of Tumor Markers, HE4 measurements, either alone in combinations with CA125 in ROMA, should be considered for differential diagnosis of pelvic masses, especially in premenopausal patients (*40*). Recently, urine has been extensively investigated as non-invasive alternative for HE4 detection.

## Human epididymis protein 4 in urine

As a small protein with a molecular weight below the glomerular filtration cut-off, HE4 is excreted and detected in urine (*51*-*53*). It was first detected in 2010 by Hellström *et al.* in urine of patients with OC (*54*). It has been shown that serum *vs.* urine have comparable ability to detect OC and discriminate cancer from healthy or benign conditions (*18*, *20*, *52*). Indeed, urinary HE4 has similar sensitivity and specificity as serum HE4 (*52*). However, it appears that concentrations of HE4 in urine are significantly higher than in blood, potentially due to the excretion and accumulation in urine, with HE4 in serum ≥ 70 pmol/L *vs.* compared to urine ≥ 13,600 pmol/L in OC patients (*36*, *55*-*57*). Therefore, increase in the urine HE4 could be detected earlier in the course of the disease, potentially improving OC prognosis (*18*). Indeed, higher HE4 concentrations were found in > 70% of early stage (I//II) OC patients (*54*). Moreover, using urine could facilitate serial measurements in a follow-up or in monitoring the response to therapy as urine sampling can be performed any time, and is not limited with health status of oncology patients as blood sampling can be (*18*, *53*). Indeed, urine HE4 protein is easily accessible and non-invasive marker that can provide important clinical information (*18*, *54*-*57*).

## Laboratory aspect and methodology of urine HE4 detection

### Preanalytical aspect of HE4 detection in urine

The well-standardized procedures for collection, transport, and sample preparation are the basis of an effective diagnostic strategy for the analysis of HE4 in urine (*58*, *59*). Preanalytical factors such as urine collection could impact the measurements, whether a portion or 24-hour sample is used, or the time of day the sample is taken (*58*, *59*). In all studies reported, HE4 protein has been measured in single-voided portion of urine in dry sterile collection pots. Where reported, for OC patients HE4 was assayed in first-morning urine collected prior to surgery or any clinical interventions, while for healthy donors it was random portion (*18*, *54*, *57*). The 24-hour urine specimens were not used, probably due to the inconvenience of collection. According to the guidelines by European Federation of Clinical Chemistry and Laboratory Medicine (EFLM), protein concentration in single-voided urine samples should be reported with creatinine (CRE), which reflects urine volume (*59*). In studies investigating diagnostic value of urine HE4, some measured urine HE4 protein alone, while others employed ratio between urine HE4 protein and urine creatinine (HE4/CRE) (*18*, *54*, *56*, *57*).

Human epididymis protein 4 has been shown as stable marker, detectable in blood up to 7 days after collection and storage at 4 °C (*60*). Short-term storage of HE4 in urine has not yet been investigated. When stored at - 80 °C, HE4 can be detected in both blood and urine for several months after collection (*18*, *60*). Additionally, the processing of urine sample play a role in obtaining the accurate and reliable results (*58*, *59*). Human epididymis protein 4 was detected in diluted urine in ratio 1:40 (*18*, *54*) and 1:100, using the diluent solution provided by manufacturers of the assay (*55*, *57*). Some authors do not disclose information even if dilution was performed, or dilution is variable in each patient. Dilution of urine is required as concentration of HE4 protein in urine of OC patients are expected to be high (> 13,600 pmol/L), which is above the range of detection of the available HE4 tests (*55*, *56*). It was observed that urine diluted minimally 1:40 is required to obtain signals without high urine background in standard immunoassays (*18*, *54*). Herein, we would like to highlight the need for better reporting the preanalytical data, as many researchers do not disclose sufficient information (*60*). Therefore, the knowledge on the preanalytical factors affecting the HE4 detection in urine are limited and must be further investigated.

### Analytical aspect of HE4 detection in urine

Most commonly employed methods of HE4 detection and quantification in all body fluids are immunoassays (*61*, *62*). First HE4 detection method was manual enzyme-linked immunosorbent assay (ELISA) (Fujirebio Diagnostics Inc., Malvern, USA), and is employed in majority of studies using urine (*31*, *54*). It is currently considered method of choice for detecting HE4 from urine specimens. However, automated platforms have been introduced since, leading to improved efficiency and accuracy of laboratory HE4 testing (*61*, *62*). Moreover, they are available in clinical settings (*61*, *62*). Analyzers that were used for HE4 detection from serum and urine include a two-step non-competitive chemiluminescence microparticle immunoassay (CMIA) (ARCHITECT HE4, Abbott Laboratories, Chicago, USA); a two-step competitive sandwich chemiluminescence enzyme immunoassay (CLEIA) (Lumipulse G600II, Fujirebio Diagnostics Inc., Malvern, USA); and one-step sandwich electrochemiluminescence immunoassay (ECLIA) (Cobas Elecsys HE4, Roche, Basel, Switzerland) (*55*, *57*, *63*).

The comparison of automated HE4 methods to ELISA using serum revealed that performances for detection of OC in woman with adnexal masses are comparable with the reference ELISA and can be used in practice (*61*, *62*). Therefore, all three methods can be used as a second choice depending on the availability in the laboratory. Such method comparison for urine specimens are yet to be investigated. The immunoassay used should always be reported together with HE4 marker results (*61*, *62*). When measuring HE4/CRE, creatinine is measured in same urine portion using a commercially available kits following manufacturer protocol (*18*, *54*). Then, urine HE4 data measured by immunoassays is normalized by calculating the ratio between HE4 (pmol/L)/CRE (mmol/L). The HE4/CRE is reported as numerical value without unit (*18*, *54*).

Mass spectrometry is another approach for HE4 determination in urine (*64*). Previously, HE4 protein was found elevated in urine of OC patients, together with various proteins, showing that urine can have proteomic signature that can discriminate malignant patients from individuals with benign disease (*64*).

Furthermore, Point-of-Care (POC) diagnostics are emerging as promising platforms for on-site tumor marker detection, as they can make testing process faster, easier, cost-effective (*65*). Recently, two POC tests were developed for HE4 detection from urine (*66*, *67*). A novel test that combines lateral flow technology and cell phone analysis was developed to measure HE4/CRE ratio in urine for monitoring the disease recurrence (*66*). However, it has not been tested yet in human samples (*66*). Another POC for urinary HE4 detection was developed based on sandwich ELISA on a microchip and analysis using cell phone (*67*). The colorimetric reaction is imaged using a cell phone, and the HE4 concentration is reported on the cell phone screen (*67*). This POC device was tested in urine of OC patients and achieved sensitivity of 90% and a specificity of 90% for OC diagnosis (*67*).

The summary of the detection of HE4 in urine and corresponding methodology are described in the Table 1.

**Table 1:** Detection and quantification of human epididymis protein 4 and human epididymis protein 4 to creatinine ratio in urine for ovarian cancer diagnosis

**Urine tumor marker**	Urine dilution*	**Urine HE4 detection method**	**HE4 test manufacturer**	**Range of urine HE4 (pmol/L)**	**Number of subjects studied in the study**	**Cut-off value**	**Specificity of the test for the presence of ovarian cancer (%)**	**Sensitivity of the test for the presence of ovarian cancer (%)**	**Reference**
HE4	n/a	Mass spectrometry	n/a	n/a	20 OC	n/a	n/a	n/a	(64)
HE4	1:20	Microchip ELISA	n/a	43.8-2808.6	19 OC 20 HC	43.8 pmol/L	90	84	(67)
HE4	n/a	ELISA	Fujirebio Diagnostics Inc., USA	n/a	72 OC 160 BC	19,161 pmol/L	90	65	(56)
HE4	1:100	CMIA	Abbott Laboratories, USA	n/a	23 OC 37 BC 18 HC	13,000 pmol/L	75	78	(55)
HE4	n/a	ECLIA	Roche Diagnostics, Shanghai, China	15-1500	31 OC 36 HC	14,116 pmol/L	100	84	(63)
HE4	1:100	CLEIA	Fujirebio Europe N.V., Belgium	600-1,717,000	17 OC 51 BC 46 HC	9100 pmol/L	71	59	(57)
HE4/CRE	1:40	ELISA	Fujirebio Diagnostics Inc., USA	10-10,000	79 OC 20 BC 36 HC	n/a	100	73 (for OC I/II) 88 (for OC III/IV)	(54)
HE4/CRE	n/a	ELISA	Fujirebio Diagnostics Inc., USA	n/a	72 OC 160 BC	152.5^†^	93	71	(56)
HE4/CRE	1:40	ELISA	Fujirebio Diagnostics Inc., USA	n/a	92 OC 82 BC 60 HC	3.5^†^	95	98	(18)
HE4/CRE	n/a	ELISA	Fujirebio Diagnostics Inc., USA	0-25,000	22 OC 10 BC 11 HC	0.89^†^	95	68	(19)
The table presents the summary of all published studies of HE4 detection in single-voided urine in healthy individuals and ovarian cancer patients with corresponding methodology and clinical samples. *The urine is diluted with commercial diluent solution provided by the manufacturer of each assay. **^†^**HE4/CRE is reported without units and is calculated by HE4 in pmol/L and CRE in mmolL. n/a - not applicable (not reported). HE4 - Human Epididymis protein 4. CRE - Creatinine, determined in same urine portion by standard biochemistry methods, needed for the normalization of urine volume. ELISA - Enzyme-linked immunosorbent assay. CMIA - Chemiluminescence microparticle immunoassay. ECLIA -Electrochemiluminescence immunoassay. CLEIA - Chemiluminescence enzyme immunoassay. OC - Ovarian cancer. OC I//I - Ovarian cancer early stages I and II. OC III/IV - Ovarian cancer advanced stages III and IV. BC - Benign conditions. HC - Healthy controls.									

### Postanalytical aspect of HE4 detection in urine

Understanding the normal range and the factors affecting HE4 concentration enable proper interpretation of results (*68*, *69*). The biological factors that can affect HE4 concentration are age and menopausal status, with the increasing concentration with age (*69*). The HE4 serum concentration in healthy women range from 60 pmol/L to 150 pmol/L with the relationship between HE4 serum concentration and increasing age (*50*). Therefore, cut-off is often clinically considered for serum HE4 > 60 pmol/L for premenopausal and > 170 pmol/L for postmenopausal women (*50*). In most literature, an upper limit of normal serum HE4 threshold is considered 70 pmol/L, as it has been validated in healthy women (*37*). For urine, different cut-off values between healthy and cancer, independent of menopausal status, have been reported and include urine HE4 concentration > 9100 pmol/L, > 13,000 pmol/L, or > 19,161 pmol/L for OC (*55*-*57*). For HE4/CRE ratio same is observed, with very wide range of the cut-off of HE4/CRE > 0.89, > 3.5, and > 152.5 (*18*, *19*, *56*). Herein, we have calculated the average of the following studies and consider that urine HE4 concentration ≥ 13,600 pmol/L and HE4/CRE ≥ 52 are present in urine of OC patients. However, based on current knowledge, the appropriate cut-off values are yet to be determined.

Other pathophysiological factors that affect HE4 concentration are presence of other malignancies and renal diseases (*69*-*72*). Human epididymis protein 4 concentration should be interpreted carefully in patients with endometrial cancer (EC), in order to avoid false-positive results (*72*). Moreover, urine HE4 may be affected by renal function, as glomerular filtration rate decreases with age (*69*). Older woman with decreased glomerular filtration are expected to have higher serum HE4 and lower urine HE4 concentrations (*63*, *69*). Indeed, serum HE4 concentration is significantly increased in the patients with chronic kidney disease (CKD) or renal failure, with serum HE4 > 2000 pmol/L (*70*, *71*). However, in those patients, urine HE4 could be a potentially more reliable test for OC (*63*). Recently, Fan *et al*. compared urine HE4 concentrations in patients with OC, CKD and healthy individuals (*63*). The urinary HE4 concentrations were significantly higher in patients with OC compared to CKD and healthy individuals (*63*). Moreover, there was no significant difference between healthy and CKD patients (*63*). However, more studies are needed to validate those findings. Therefore, urine HE4 concentrations should be interpreted carefully together with other clinical and biochemical parameters.

## Potential clinical applications of urine HE4 in ovarian cancer

### The role of urine HE4 in the differential diagnosis

Urine HE4 concentration and HE4/CRE ratio are investigated as a tools for differential diagnosis in woman with pelvic masses (*18*, *19*, *21*, *54*). In a study by Hellstrom *et al*., HE4 was measured by ELISA and reported as HE4/CRE ratio (*54*). At the specificity of 100%, 73.3% of early stage OC (I//II) patients and 87.5% of advanced stages OC (III/IV) had higher HE4/CRE values (*54*). Indeed, in a study by Liao *et al*. when HE4 was measured by ELISA, using ratio HE4/CRE could discriminate between cancer and healthy controls (*18*). Urine HE4 from women with serous OC had higher HE4 concentrations than other subtypes of OC (*18*). The serum and urine samples obtained on the same day were tested for HE4 and achieved similar results, suggesting that urine can be used as a non-invasive alternative for HE4 testing (*18*). In a study by Stiekema *et al*., higher urine HE4 was found by ELISA in OC patients, and using HE4/CRE ratio could differentiate healthy or benign controls from epithelial OC (*19*). Another pilot study demonstrated the utility of urine HE4 concentration alone measured by ELISA and HE4/CRE ratio as potential tumor markers for the detection of OC (*56*). The HE4/CRE ratio appeared to be better than just HE4 for the pathological diagnosis of epithelial OC (*56*). In study by Fan *et al*., diagnostic value was studied for serum, urine HE4 and urine-to-serum HE4 ratio using ECLIA (*63*). Urinary HE4 concentration could discriminate OC patients from healthy controls and patients with CKD, with good diagnostic accuracy with specificity of 100% and sensitivity of 84% (*63*). In fact, urine HE4 alone achieved higher sensitivity and specificity than serum HE4 or urine-to-serum HE4 ratio (*63*). In a study by Mackus *et al*., urine HE4 measured by CMIA was higher in patients with OC when compared to healthy individuals (*55*). Recently, in a study by Barr *et al*., HE4 and CA125 were measured by CLEIA from self-sampled urine in symptomatic women of suspected malignancy (*57*). Both HE4 and CA125 were higher in urine of patients with OC, when compared to benign conditions or healthy individuals (*57*). CA125 was found present in higher concentration in serum, with average of 14.3 U/mL, compared to urine with average concentration of 3.3 U/mL, respectively (*57*). On the contrary, HE4 was found higher in urine, with average concentration of 8415 pmol/L, compared to serum where average concentration was 79 pmol/L (*57*). Urine HE4 concentrations could predict women with OC, with higher urine HE4 (> 9100 pmol/L) in 71% of women presented with OC (*57*). Moreover, the high urine HE4 concentrations were detected in early-stage OC (I/II) (*57*). The diagnostic performance reached sensitivity of 71% and specificity of 59%, respectively (*57*).

A recent meta-analysis involving total of 649 patients determined the pooled diagnostic accuracy of urine HE4 (using together HE4 and HE4/CRE as single marker entity called “urine HE4”) with a sensitivity of 80% and specificity of 93% for detecting OC, showing it to be a useful tool in differential diagnosis of OC (*21*).

### The role of urine HE4 in the chemotherapy response

It has been previously reported that overexpression of HE4 promotes chemotherapy resistance (*33*, *73*). Indeed, the preoperative serum HE4 has a good predictive value for platinum-based chemotherapeutic resistance (*45*, *74*). In a study by Liao *et al*., serial measurements by ELISA of serum CA125, serum HE4 and urine HE4 in patients with OC revealed that blood concentrations for both CA125 and HE4 decrease in response to primary surgery and chemotherapy, irrespective of platinum response status (*18*). Human epididymis protein 4 concentration in urine employed as HE4/CRE ratio remained higher in patients who proved to be platinum resistant, while decreased urine HE4 concentration and HE4/CRE ratio were observed in patients who were platinum sensitive, thus indicating that urine HE4 could be a useful tool for early identification of platinum resistance (*18*).

### The role of urine HE4 in the detection of recurrence

Despite all the efforts, OC relapse occurs in 70% of the patients within the first 3 years (*6*). In a study by Liao *et al*., serum CA125 and HE4, and urine HE4 (as HE4/CRE ratio) were assessed by ELISA in patients who achieved clinical remission after primary treatment (*18*). The patients were followed longitudinally with serial measurements of serum CA125 and urine and serum HE4 for mean 38 months after diagnosis (*18*). In three patients, urine samples repeatedly had higher HE4/CRE values (HE4/CRE > 3.5), while the serum samples were normal for both HE4 and CA125 (*18*). When these patients subsequently developed clinically detectable OC recurrence, serum HE4 and CA125 concentrations became higher again, together with urine HE4/CRE ratio (*18*). Therefore, urine HE4/CRE was elevated earlier before other clinical or biochemical signs of relapse, proving it to be a useful marker in the follow-up and detection of recurrence (*18*).

### The role of urine HE4 in detection of low-malignant-potential ovarian tumors

One problem associated with the preoperative evaluation of pelvic masses concerns the identification of low-malignant-potential (LMP) tumors (*75*). These tumors are neoplasms in which abnormal cells form in the tissue covering the ovaries, and can transform into cancer, but usually do not (*75*). Therefore, LMP tumors are associated with a good prognosis (*75*). However, the minority of the patients can develop a more progressive malignant disease (*75*). Previously, higher HE4 protein was detected by ELISA in urine of women with LMP tumors, and expressed as HE4/CRE ratio (*18*). In fact, the detection of HE4 in this particular type of tumor was found to be better in urine than in concomitantly obtained serum samples from same patients on the same day which did not differ from healthy controls (*18*). The overall sensitivity of HE4/CRE in LMP samples was ranging from 73% to 80% (*18*). Further studies are needed to learn whether the women with LMP tumors who have elevated HE4 urine concentration or HE4/CRE ratio are at greater risk to later develop more progressive cancer (*18*).

### Urine HE4 in other cancers

Increased HE4 tissue or serum expression has been demonstrated in other malignant neoplasms, including, endometrial, breast, lung and gastric cancers (*30*, *72*, *75*, *76*). The overexpression in > 90% of EC has sparked interest in its potential utility as a diagnostic marker for this disease (*46*, *77*, *78*). Indeed, serum HE4 concentrations (> 70 pmol/L) are present in 63% of EC patients (*37*). Recently, in a study by Njoku *et al*., urine HE4 and CA125 were analyzed by CLEIA in attempt to find a simple urine-based test to triage symptomatic women for EC (*79*). Human epididymis protein 4 concentration in urine was significantly higher in women with EC compared to those without cancer (*79*). However, urine HE4 had a weaker diagnostic value than urine CA125 in detection of EC (*79*). The use of urine HE4 in other malignancies was not yet evaluated. Therefore, up to this date, urine HE4 still stays most promising marker for detection of OC.

## Discussion

The absence of precise diagnostic tools for OC is a important factor responsible for the poor prognosis and high mortality (*6*, *11*). Recently, there has been a growing interest in using urine for detecting OC (*14*-*16*). In this review, we presented the recent advancements of the diagnostic roles and laboratory aspect of urine HE4 protein in clinical management of OC. Elevated HE4 protein was found in patients with OC in numerous independent studies (*18*, *19*, *54*-*57*). Current gold standard CA125 is a 1-5 MDa protein not expected to pass freely in urine in patients whose renal function is maintained (*20*, *80*). However, detected CA125 could be present in fragments, as a result of proteolytic degradation (*80*). Previously, CA125 had very low specificity < 40% for detecting OC, even when combined with other markers (*53*). However, it has been evaluated recently in woman with suspected malignancy, and achieved sensitivity of 71% and specificity of 74% for detection of OC (*57*). Therefore, it needs to be further investigated as urine target.

Urine HE4 is a tumor marker used as non-invasive alternative to serum HE4 in detection or management of OC (*18*, *54*-*57*). It can stratify OC patients from healthy or benign gynecological conditions (*18*, *54*-*57*, *63*). Indeed, urine HE4 concentration was detected elevated earlier than serum HE4 when following disease progression, and could detect recurrence prior to clinical determination of relapse (*18*). Moreover, it was shown useful as predictor of therapy resistance (*18*).

Furthermore, we evaluated the laboratory aspects of HE4 detection in urine. When choosing a target marker, we recommend measuring urinary HE4/CRE ratio, rather than urinary HE4 alone. Assessments of protein-to-creatinine ratios in single voided samples have replaced most of the timed collections in the diagnostics or follow-up of patients (*59*). As HE4 measurements are impacted by the volume of urine, it is recommended by EFLM to report protein concentrations together with CRE to normalize the urine volume and compensate for differences in intra-individual diuresis (*59*). Moreover, in some cases, HE4/CRE ratio was more sensitive and specific for pathological diagnosis of OC, than unchanged urine HE4 concentration (*56*).

As previously explained, we observed high variability in preanalytical factors, such as urine collection and processing (*18*, *19*, *54*-*57*). Therefore, it would be necessary to standardize the way urine is processed prior to clinical use (*58*). Moreover, there is currently no consensus on the cut-off between healthy and OC patients, with different value in each study (*54*-*57*). Usually, the analytical method gives a cut-off value depending on the pre-test probability of the disease (*81*). In the absence of any previous information or test results in a person, the pre-test probability can be estimated as the prevalence of the disease (*81*). Therefore, the cut-off value is not universal and will depend on the prevalence of OC in specific population, country or region (*81*). There have been proof that higher cut-off can improve specificity for detection of OC, as seen in Liao *et al*., where specificity of 95% was achieved at the cut-off value of urine HE4/CRE ratio of 3.5, while specificity of 98% was observed at the higher cut-off of HE4/CRE ratio of 5.0, respectively (*18*). At this moment, it is still left to the decision of laboratory specialists and clinicians. More studies are needed to determine the optimal cut-off value between healthy and OC. Overall, urine HE4 and HE4/CRE ratio have shown as diagnostic and prognostic markers in management of woman with suspected OC. However, bigger cohorts on patients are needed to validate those findings. More effort of factors affecting urine HE4 concentration and standardization of HE4 testing are needed prior to implementation in clinical setting.

## Conclusions

Urine HE4 concentration and HE4/CRE ratio are alternative non-invasive markers for OC that can discriminate cancer from healthy individuals or benign conditions (*18*, *54*-*57*, *63*). When compared to serum, urine HE4 is present in higher concentrations, which could facilitate detection and achieve earlier diagnosis (*55*, *57*, *63*). Moreover, it has been demonstrated to be a useful predictive tumor marker for detection of chemotherapy resistance and disease recurrence (*18*). However, it is important to standardize the preanalytical phase and choice of HE4 detection method using urine. Additionally, it is necessary to establish appropriate cut-off values between cancer and healthy, and investigate the factors that can impact urine HE4 concentration prior to implementation in the clinical practice.

## Data Availability

No data was generated during this study, so data sharing statement is not applicable to this article.
